# The Possible Relationship between the Abuse of Tobacco, Opioid, or Alcohol with COVID-19

**DOI:** 10.3390/healthcare9010002

**Published:** 2020-12-22

**Authors:** Yusuf S. Althobaiti, Maram A. Alzahrani, Norah A. Alsharif, Nawal S. Alrobaie, Hashem O. Alsaab, Mohammad N. Uddin

**Affiliations:** 1 Addiction and Neuroscience Research Unit, Health Science Campus, Taif University, Taif 21974, Saudi Arabia; S43503198@students.tu.edu.sa (M.A.A.); S43503701@students.tu.edu.sa (N.A.A.); S43500511@students.tu.edu.sa (N.S.A.); 2 Department of Pharmacology and Toxicology, Health Science Campus, College of Pharmacy, Taif University, Taif 21974, Saudi Arabia; 3 General Directorate of Narcotics Control, General Administration for Precursors and Laboratories, Ministry of Interior, Riyadh 11134, Saudi Arabia; 4 Department of Pharmaceutics and Pharmaceutical Technology, Taif University, Taif 21944, Saudi Arabia; h.alsaab@tu.edu.sa; 5 College of Pharmacy, Mercer University, Atlanta, GA 30341, USA; Uddin_mn@mercer.edu

**Keywords:** substance use disorder, COVID-19, smoking, opioids, alcohol

## Abstract

*Introduction:* Substance use disorder has been frequently reported to increase the risk of infectious diseases, which might be owing to the sharing of contaminated inhalation, smoking, vaping, or injection equipment. *Aim:* This review analyzes the recent literature with the aim to put in light the possible relationship between the abuse of different substances (Tobacco, opioid, and Alcohol) with coronavirus disease (COVID-19). *Tobacco:* Multiple studies confirmed that cigarette smoking affects the respiratory system by increasing the expression of angiotensin-converting enzyme-2 (ACE2) receptors, which have a significant association with COVID-19 infection rate and disease severity. *Opioid:* Studies conducted regarding the association of opioid use disorder (OUD) and COVID-19 infection severity are limited; however, opioids can lead to both respiratory depression and kidney injuries, causing poor prognosis for those with COVID-19 infections. *Alcohol:* People with alcohol use disorders are at risk of developing acute lung injury and severe COVID-19 infection. Alcohol consumption during the COVID-19 pandemic has two possible scenarios: either increased or decreased based on situations. *Conclusion:* SUD has been frequently reported to have a positive relationship with COVID-19 severity Further studies are needed to understand the effects of opioids and alcohol abuse on COVID-19.

## 1. Introduction

Coronavirus disease 2019 (COVID-19) caused by a recently discovered coronavirus [1], can cause potentially severe acute respiratory infections that might lead to death [2]. The first case of COVID-19 was reported from Wuhan, China, in early December 2019 [3]. Within a month, COVID-19 was declared pandemic due to its rapid transmission across continents, with the number of cases and deaths rising daily [1]. Although most infected individuals exhibit mild illness (81%), 14% have serious symptoms, while 5% have a critical condition. Approximately including invasive ventilation due to acute respiratory distress syndrome [4]. The most common and apparent symptoms for mild to moderate cases are fever, dry cough, and tiredness. In critical cases, the symptoms are severe, such as shortness of breath, movement or speech loss, and chest pain [1]. On average, symptom onset may be approximately 5–6 days; however, symptoms can take up to 14 days to appear [1]. Older patients, as well as patients with underlying medical conditions have been reported to have a higher risk for severe illness with coronavirus [5].

Substance use disorders (SUD) have been frequently reported to increase the risk of infectious diseases [6]. This increased risk of infectious disease among those addicted to drugs might be owing to the sharing of contaminated inhalation, smoking, vaping, or injection equipment. SUD is among the most prevalent psychiatric disorders worldwide. It is a chronic brain disorder that causes powerful physical and psychological cravings for mind-altering substances [7]. This addiction has several reasons, such as pleasure-seeking, peer-pressure, performance improvement, and self-medication for a preexisting mental disorder [8]. During COVID-19, these substances had reported significant changes in consumption status as a result of pandemic related restrictions. SUD’s major manifestation is compulsive drug use despite severe harmful complications, such as failing in essential tasks, medical illness, or involvement in criminal activity for supporting the addictive behavior [9,10].

Previous studies assumed an increase in the substance of abuse consumption during the COVID-19 related lockdown compared to the period before the lockdown. This review analyzes the recent literature with the aim to put in light the possible relationship between the abuse of different drugs and coronavirus disease (COVID-19).

## 2. Substances Used Disorders (SUD) and COVID-19

### 2.1. Tobacco Uses and COVID-19

Cigarette smoking can affect multiple organs, thereby leading to different diseases, and in general, reduces the overall health of smokers [11]. This section discusses different views of researchers on the relationship of tobacco smoking with the severity of COVID-19 infection. A review of studies by public health experts convened by WHO on 29 April 2020 found that smokers are more likely to develop severe disease with COVID-19, compared to non-smokers [12]. Furthermore, Vardavas and Nikitara et al. recent systematic review [12] on five studies [3,13,14,15,16] concluded that “smoking is most likely associated with negative progression and adverse outcomes of COVID19”.

The largest study population of 1099 patients with COVID-19 was provided by Guan et al. [3] from multiple regions of mainland China. Descriptive results on the smoking status of patients were provided for the 1099 patients, of which, 926 had non-severe symptoms and 173 had severe symptoms Among the patients with severe symptoms, 16.9% were current smokers and 5.2% were former smokers, in contrast to patients with non-severe symptoms where 11.8% were current smokers and 1.3% were former smokers.

Another study by Zhou et al. [15] studied the epidemiological characteristics of 191 individuals infected with COVID-19, without, however, reporting in more detail the mortality risk factors and the clinical outcomes of the disease. Among the 191 patients, there were 54 deaths, while 137 survived. Among those that died, 9% were current smokers compared to 4% among those that survived, with no statistically significant difference between the smoking rates of survivors and non-survivors (*p* = 0.21) with regard to mortality from COVID-19. Similarly, Zhang et al. [13] presented clinical characteristics of 140 patients with COVID-19. The results showed that among severe patients (*n* = 58), 3.4% were current smokers and 6.9% were former smokers, in contrast to non-severe patients (*n* = 82) among which 0% were current smokers and 3.7% were former smokers, leading to an OR of 2.23; (95% CI: 0.65–7.63; *p* = 0.2).

Huang et al. studied the epidemiological characteristics of COVID-19 among 41 patients [17]. In this study, none of those who needed to be admitted to an ICU (*n* = 13) was a current smoker. In contrast, three patients from the non-ICU group were current smokers, with no statistically significant difference between the two groups of patients (*p* = 0.31), albeit the small sample size of the study. Finally, Liu et al. found among their population of 78 patients with COVID-19 that the adverse outcome group had a significantly higher proportion of patients with a history of smoking (27.3%) than the group that showed improvement or stabilization (3.0%), with this difference statistically significant at the *p* = 0.018 level [14]. In their multivariate logistic regression analysis, the history of smoking was a risk factor of disease progression (OR = 14.28; 95% CI: 1.58–25.00; *p*= 0.018) as shown in Table 1.

**Figure 1:**
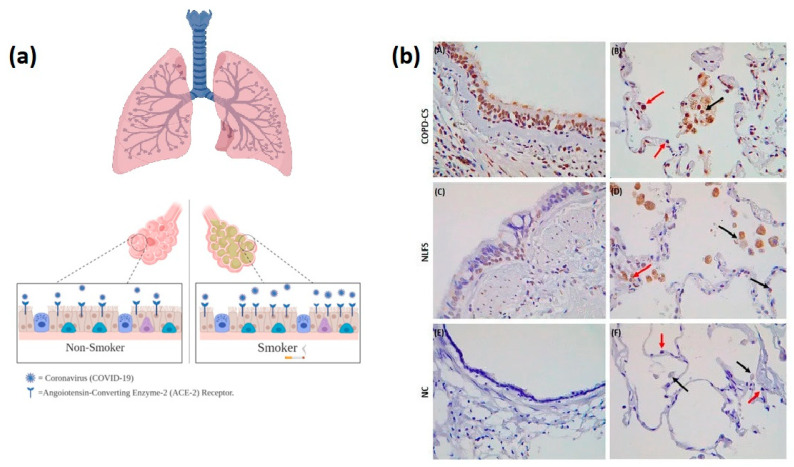
(**a**) Effect of smoking on angiotensin-converting enzyme-2 (ACE) receptors and its relationship to COVID-19: smoking may increase the number of ACE receptors, which leads to an increase in the severity and rate of COVID-19 infection. (**b**) The first immunohistochemical human lung evidence for ACE2 receptor expression in smokers and patients with chronic obstructive pulmonary disease (COPD). Current smoker with COPD, (**A**) indicating positive staining in the small airway epithelium but also apical including cilia (**B**) red arrows showing positive staining in type-2 pneumocytes and black arrows indicating alveolar macrophages positive for the ACE2 receptor. Normal lung function smoker (NLFS), (**C**) and (**D**) representing similar pattern for COPD although a little less staining is observed. Normal controls (NC), (**E**) and (**F**) no staining observed in any of the areas. Taken with permission from [10].

The risk factor relation attributed to that Smoking has been reported to increase the expression of angiotensin-converting enzyme-2 (ACE2) receptors, which has been known to be a target for the severe acute respiratory syndrome coronavirus 2 (SARS-CoV-2) and human respiratory coronavirus as shown in Figure 1, which leads to an increase in the severity of COVID-19 Symptoms [18]. Another study by Vanderbruggen et al. confirmed an increase in alcohol consumption and cigarette smoking during the COVID 19 related lockdown compared to the period before the lockdown. A total of 3632 respondents (mean age 42.1 ± 14.6 years; 70% female) filled out the survey. Overall, respondents reported consuming more alcohol (*d* = 0.21) and smoking more cigarettes (*d* = 0.13) than before the COVID-19 (both *p* < 0.001) [19]. The odds of smoking more cigarettes during the lockdown were associated with younger age, current living situation, lower education, lack of social contacts, Boredom, reward after a hard-working day, loss of daily structure, loneliness, and conviviality were the main reasons for consuming more of the various substances. Conversely, Lippi and Henry’s meta-analysis reported no smoking status associated with the severity of COVID-19 [20]. The studies conducted by Han et al. and Yuan et al. concluded that in chronic cigarette smoke-induced pulmonary arterial hypertension in rats and angiotensin II levels in the lungs were increased with an increased expression of ACE and decreased expression of ACE2 [18,21].

Interestingly, smoking could be a potential factor for the doubled death rates observed in men than women due to COVID-19. A study of 140 patients with COVID-19 conducted in China [14] found that the sex distribution was equal, while in another study of critically ill patients, [22] more men were affected (67%) than women (33%). This might be due to smoking as men smoke more than women in China (smokers in 2018, 288 million men vs. 126 million women) [23].

### 2.2. Opioid Used Disorders and COVID-19

Unlawful opioid use has a significant impact on society, including increased mortality and morbidity, marginalization, and criminal behaviors, including the enormous black-market economy from opiate trafficking [24]. Opioid abuse involves longstanding changes in the mesolimbic dopaminergic system, involving a tolerance of the euphoric effects (liking) and drug sensitization for the urge to use drugs (wanting) [25]. In 2014, approximately 435,000 Americans aged 12 or older reported using heroin, and 4.3 million reported nonmedical use of prescription opioids [26].

Studies conducted regarding the association of opioid use disorder (OUD) and COVID-19 infection or its severity are limited. A review paper explained how opioids could increase the severity of COVID-19 infection, with its effect on different body systems leading to complications [27]. Opioids such as methadone are pharmacologically respiratory depressants that cause respiration disruption; patients often very slowly and incompletely develop tolerance to methadone and other opioids. Thus, COVID-19 patients undergoing Methadone maintenance treatment should be monitored closely for worsening respiratory functions [28]. Usage of opioids leads to multiple and complex interactions within different body systems, especially the endocrine and nervous systems simultaneously, which involves alterations in the autonomic nervous system (sympathetic and parasympathetic. Given the high prevalence of kidney impairment in hospitalized COVID-19 patients, the effects of opioids on renal function might increase the risk of hospital death [29].

It is necessary to note that opioids are usually used safely in anesthesia and as a pain killer in the perioperative period in patients with kidney disease. The renal toxicity appears owing to the improper use of opioids: accidental higher doses, in the presence of other toxins, with preexisting dehydration, or prostate enlargement [30]. Constant use of opioids as noted by Novick et al. [31], appears to have a higher incidence of toxicity owing to the accumulation of metabolites, which could cause undesired side effects. Overdose of opioids can result in acute kidney injury (AKI) owing to different mechanisms and causes, such as dehydration, hypotension, rhabdomyolysis, and urinary retention [32]. One study that assessed the relationship between AKI and COVID-19 found that AKI occurs early and in temporal association with the failure of respiration, and it is associated with a bad prognosis [33]. Opioids can lead to both respiratory depression and kidney injuries, causing poor prognosis for those with COVID-19 infections.

Chronic kidney disease may result owing to the method of drug administration: skin popping resulting in amyloidosis. Heroin-associated nephropathy is now considered to be linked to a toxin introduced into heroin during drug processing [34]. One recent study reported that early identification of patients at risk in need of immediate appropriate support and avoidance of nephrotoxins might help in improving the prognosis of patients with COVID-19 infection [34].

Elderly patients have higher risks, as age-related changes can alter opioid pharmacokinetics, thereby resulting in undesired side effects. Reduced organ function of the liver and kidney, alterations in adipose tissue composition, and altering opioid pharmacokinetics allow metabolites to accumulate and exist for a longer duration [35]. The actual incidence of renal failure from opioid use is not well-defined owing to under-recognition and under-reporting. The common mechanism for AKI with opioid use is in the context of multi-organ failure from respiratory depression, hypoxia, and volume depletion with or without rhabdomyolysis [36].

There are nearly 20 identified opioid peptide receptors that can be activated by endorphins [37]. The activation of opioid peptide receptors has been shown to inhibit the cardiac excitation-contraction process and decrease the arterial blood pressure in normotensive male Sprague-Dawley rats. The PNS effect will increase, which will counteract the SNS, thereby leading to decreased heart rate and blood pressure [38]. Similarly, opioid anesthetics have been demonstrated to reduce the renal blood flow when compared with other anesthetics, such as ketamine [39].

Another study also confirmed that alcohol, amphetamine, and cocaine abusers are more likely to suffer from renal failure [40,41,42]. Furthermore, we already know that comorbidities increase the risk of severe infections, such as cardiac diseases, resulting from the overuse of alcohol, heroin, cocaine, or amphetamine [43,44,45,46]. However, the direct relationship between OUD and COVID-19 is not well established, but according to the previous reviewed reports, attention should be given to people with a history of OUD or any patients with comorbidities such as chronic kidney disease; physicians should consider the consequences of opioid over-usage when treating patients to avoid complications and bad prognosis.

### 2.3. Alcohol Used and COVID-19

**Figure 2:**
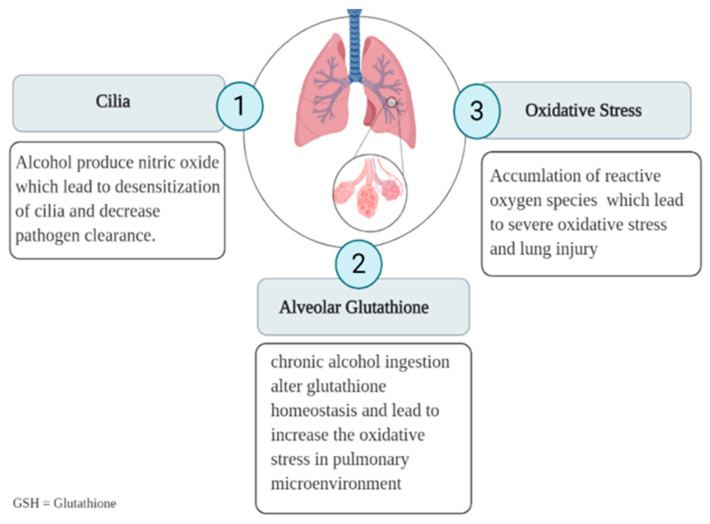
Potential mechanisms by which alcohol might cause lung injury: chronic alcohol consumption might produce nitric oxide, which can lead to desensitization of cilia and decrease pathogen clearance (**1**), disturb glutathione homeostasis and cause oxidative stress (**2**), and lead to the accumulation of reactive oxygen species (**3**).

Alcohol use disorder (AUD) is a chronic and relapsing disorder [47]. People with AUDs are at risk of developing acute lung injury and acute respiratory distress syndrome [48]. They are also at risk of developing severe COVID-19 infections and superinfections. The potential mechanisms by which alcohol causes lung injury starts with the upper respiratory airways as shown in Figure 2. When alcohol metabolizes, nitric oxide (NO) is produced, and the accumulation of NO may deteriorate endothelial function as may cause desensitization of cilia that can affect the pathogen clearance [49,50]. In the alveolar spaces, chronic alcohol ingestion alters glutathione homeostasis, which leads to an increase in the oxidative stress in the pulmonary microenvironment [50,51]. Moreover, alcohol can disrupt both the innate and adaptive immune systems [52] by impairing the capacity of alveolar macrophages to phagocytose and clear bacteria [51]. Rehm et al. in a systematic review, assessed alcohol consumption behavior during the COVID-19 pandemic. They found two scenarios: the first scenario predicts an increase in alcohol consumption, and the other predicts a reduction in alcohol consumption [52]. Most governments have responded to the COVID-19 pandemic by advising the public to remain indoors and avoid unnecessary social contact [53]. A study conducted in München, Germany, found that most people following these strict policies and lockdowns are suffering from the disruption of their daily routines, isolation, social distancing, financial worries, and fear of the future [54]. These factors could trigger increased alcohol consumption as a form of self-medication; a study by Wang et al. showed that more than half of the population surveyed in China reported depression, anxiety, and stress [55]. Another survey conducted in 2001–2002 by the National Institute of Alcohol Abuse and Alcoholism reported the same idea of self-medication among people who try to cope with such stressful situations [56]. The Trier Social Stress Test conducted among 39 social drinkers found an increase in alcohol craving behaviors among those who were under stressor compared with the non-stressor group [57]. In a review by Michael et al., a “stress-response-dampening theory” has been discussed, which refers to an increase in alcohol consumption during economic crises, especially among people suffering from anxiety and stress [58]. A similar situation was observed in 2003 during the severe acute respiratory syndrome (SARS) pandemic. Two survey studies conducted in China emphasized the relationship between the psychological disorders caused by the pandemic and the increase in alcohol consumption in two different settings (Hong Kong residents who were exposed to SARS and hospital employees in Beijing who were either in quarantine or worked in high-risk hospital wards) [59,60]. In June 2020, a study was conducted among 1074 Chinese who showed an increased risk of potent psychiatric disorder with a higher rate of anxiety, depression, hazardous and harmful alcohol consumption, and lower mental wellbeing owing to the COVID-19 outbreak and mass isolation [61]. The other consequences of pandemics, such as COVID-19, include the economic ramifications that can affect alcohol users. During pandemics, people are often affected by working difficulties such as losing their jobs or reduced working hours, which can affect their total income. Therefore, a decrease in alcohol consumption and associated behaviors may occur. A similar reduction in alcohol consumption might happen due to pandemic-related restrictions, such as closing on-premises consumption [52] as shown in Table 2. Potential challenges are expected in managing patients with AUDs during this pandemic, which may worsen the treatment compliance, develop a relapse, and cause withdrawal effects [62]. Interestingly, a study that was conducted in India reported that the lockdown and closure of licensed liquor shops made alcohol abusers resorting to country/homemade liquor. These substandard and possibly contaminated liquors might cause severe health-related complications, including death [62]. With “stay home” regulations, alcohol abusers no longer have the structured time for non-alcohol-related activities, such as direct communication in social life and exercising activities, which may contribute to alcohol relapse behavior [51]. Another challenge in AUDs is during management; it is difficult to attend regular outpatient visits due to office closures, lack of public transport, or fear of infection [51,63]. Moreover, high workloads in the remaining available treatment centers, the lack of telehealth/online health service delivery, or the patients’ failure to utilize such services might lead to substandard care and poor outcomes [62]. Besides the COVID-19 protective instructions, governments should initiate public health warnings about excessive alcohol consumption during isolation [57]. A study by Matteo et al. recommended continuing group treatment sessions with alcohol abusers from their homes via available online platforms [64] and using telehealth and secure messaging services for providing alcohol counseling and addiction treatment and giving patients access to 24/7 care [51].

## 3. Possible COVID-19 Impact on SUDs

COVID-19 pandemic has affected different vulnerable health populations; one of them is patients with SUDs. There are many barriers related to substance use treatment, which already exist not only in the USA but worldwide [65]. Within this crisis, there are several obstacles, such as the challenges of the health care providers for addressing the needs of patients with OUDs in the context of longstanding rules and regulations around medications, such as buprenorphine and methadone.

Other concerns regarding SUD patients during the pandemic include following physical/social distancing in face-to-face group treatments and other mutual support groups that are critical for their recovery [65]. On 12 March 2020, after the COVID-19 outbreak was declared a pandemic by the World Health Organization, health care providers were requesting the elimination of existing barriers to treating patients with SUD. Some studies have shown the positive outcomes of telehealth while dealing with these obstacles, such as initiating and monitoring patients on buprenorphine, expanding the access to virtual support groups, and providing flexibilities in dispensing medications for OUD treatments. However, these measures could be temporary and with limited benefits in managing SUDs [66]. In addition to some concerns regarding OUD patients confronted with a crisis like COVID-19, providing health care for these patients should be a priority. These situations are similar to emergency conditions since some patients tend to inject themselves with drugs to help them cope with the crisis. Therefore, it is necessary to decrease the widespread panic and anxiety related to this pandemic during Hurricane Sandy, late October 2012 [67].

## 4. Conclusions and Future Considerations

Substances abuse might increase the risk of developing COVID-19 and the severity of this infection. Further studies are needed to address such risk and severity of COVID-19 in patients with SUD. People with a history of SUD should receive attention from healthcare providers during such pandemics which can cause stress that is one of the main reasons for relapse to drug use. Moreover, special care should be provided for current patients with SUD to make sure their therapeutic plan and medications are not affected during such pandemics. This can be done by establishing a virtual consultations and follow-up to support them and address their concerns. Group counseling and other rehabilitation programs can also be continued virtually during such pandemics to ensure drug-free lifestyle.

The relationship between smoking and the incidence of COVID-19 infection and its severity was recognized in different studies, which is a direct relationship. Patients of COVID-19 with OUD might have increased risk of worsening respiratory and renal functions especially in older patients. In addition, patients with AUD are at risk of developing severe COVID-19 infections and superinfections due to the impairments of their immune system. Further studies are needed to investigate the incidence of COVID-19 and possible complications following exposure to different drugs of abuse.

## Figures and Tables

**Table 1:** Association of smoking with negative progression and adverse outcomes of COVID19.

Authors	Setting	Study Design and Time	Sample Size	Smoking and Severity of COVID-19		
					Non-Severe	Severe
Guan et al. [3]	China	Retrospective 29 January 020	1085	Never smoked	*n* = 926	*n* = 173
					793 (86.9%)	134 (77.9%)
				Former smoker	12 (1.3%)	9 (5.2%)
				Current smoker	108 (11.8%)	29 (16.9%)
Zhang et al. [13]	China	Retrospective 16 January to 3 February 2020	140	Current smoker	*n* = 82	*n* = 58
					0 (0%)	2 (3.4%)
				Former smoker	3 (3.7%)	4 (6.9%)
Liu et al. [14]	China	Retrospective from 30 December 2019 to 15 January 2020	78	Current smoker	*n* = 67	*n* = 11
					2 (3%)	3 (27.3%)
Zhou et al. [15]	China	Retrospective multicenter cohort study until 31 January 2020	191	Current smoker	*n* = 54	*n* = 137
					5 (9%)	6 (4%)
Huang et al. [17]	China	Prospective from 16 December 2019 to 2 January 2020	41	Current smoker	Non-ICU care	ICU care
					*n* = 3 (11%)	*n* = 0

**Table 2:** Literature review of the possible scenarios of alcohol consumption behavior during the COVID19 pandemic.

Topic		Year	Author	Sitting	Finding
Increased Alcohol intake during COVID 19	1	March 2005	Lau, et al.	The study conducted among more than 800 Hong Kong residents who were exposed to the Severe Acute Respiratory Syndrome (SARS) pandemic in 2003 conducted through 2 independent telephone surveys—survey 1 were asked about SARS-related perceptions—survey 2 were asked about psychological effects of SARS such as psychosomatic problems; had increased smoking and alcohol consumption; and other.	The study found high percentages of respondents felt helpless, horrified, and apprehensive because of SARS or worried that they or family members would get the virus. Approximately half of the respondents perceived that their mental health had severely or moderately deteriorated because of the SARS epidemic. Among those who consumed alcohol, 4.7% of male respondents and 14.8% of female respondents had increased their frequency of drinking 1 year after the SARS pandemic [63].
	2	12 September 2008	Wu et al.	A survey was conducted among 549 randomly selected hospital employees in Beijing, China, concerning the psychological impact of the 2003 SARS outbreak.	The study found increase in risk of reporting psychiatric symptoms, such as alcohol abuse/dependence 3 years after the SARS outbreak among hospital employees in Beijing who were either in quarantine or worked in high-risk hospital wards, was about 1.5 times higher than for nonexposed hospital employees [64].
	3	April 1999	Sayette, et al.	A review of human studies that investigate the following hypothesis whether drinking reduces stress? (The second part of the hypothesis—i.e., stress induces alcohol consumption)	In a review by Michael et al., a “stress-response-dampening theory” has been discussed, which refers to an increase in alcohol consumption during economic crises, especially among people suffering from anxiety and stress [62].
	4	11 November 2008	Bolton et al.	A nation-wide household comorbidity survey (*n* = 43,093) conducted in 2001–2002 by the National Institute on Alcohol Abuse and Alcoholism.	The study conducted in 2001–2002 by the National Institute of Alcohol Abuse and Alcoholism reported of self-medication among people who try to cope with such stressful situations [60].
	5	June 2020	Ahmed, et al.	An online survey was conducted via Tencent on a sample of 1074 Chinese people, majority from Hubei province. To detect the mental health problems due to outbreak of the COVID-19 and mass isolation	The study was conducted among 1074 Chinese who showed an increased risk of potent psychiatric disorder with a higher rate of anxiety, depression, hazardous and harmful alcohol consumption, and lower mental wellbeing owing to the COVID-19 outbreak and mass isolation [65].
	6	6 March 2020	Wang, et al.	a cross-sectional survey design to assess the public’s immediate psychological response during the epidemic of COVID-19 by using an anonymous online questionnaire that was firstly disseminated to university students and they were encouraged to pass it on to others. Included 1210 respondents from 194 cities in China.	The study mention factors that could trigger increased alcohol consumption as a form of self-medication; more than half of the population surveyed in China reported depression, anxiety, and/or stress [59].
	7	12 September 2018	Clay, et al.	39 participants were randomly allocated to ‘stress’ and ‘no-stress’ groups; in the stress group, participants took part in the Trier Social Stress Test (TSST). Participants completed several questionnaires and computer tasks in order to assess prior alcohol use, impulsivity/risk-taking, stress-reactivity, craving and physiological biomarkers of stress. Then, participants completed a voluntary drinking task.	The Trier Social Stress Test conducted among 39 social drinkers found an increase in alcohol craving behaviors among those who were under stressor compared with the non-stressor group [61].
	8	29 April 2020	Frank, et al.	A cross-sectional evaluation sample of patients who were treated in Department of Psychiatry and Psychotherapy at München, Germany A short standardized interview was employed among 196 patients with main psychiatric diagnoses such as schizophrenia and addictive disorders. The examination included the Clinical Global Impression (CGI) Scale.	A study conducted in München, Germany, found that most people following these strict policies and lockdowns are suffering from the disruption of their daily routines, isolation, social distancing, financial worries, and fear of the future [58].
Decreased Alcohol intake during COVID 19	9	May 2020	Rehm, et al.	A systematic review of the effects of past economic crises on alcohol consumption systematic review of the effects of past economic crises on alcohol consumption. A systematic review of the effects of past economic crises on alcohol consumption systematic review of the effects of past economic crises on alcohol consumption. A systematic review of the effects of past economic crises on alcohol consumption and discussed of two possible scenarios of alcohol consumption during COVID 19 pandemic.	Jürgen et al. in a systematic review assessed the alcohol consumption behavior during the COVID-19 pandemic and found 2 scenarios: the first scenario predicts an increase in alcohol consumption and the other predicts a reduction in alcohol consumption. Increase consumption could linked to pandemic psychological effect and social restriction while decrease in alcohol intake could linked to the tight budgets and lower economic status of most population [56].
